# The UK medical education database (UKMED) what is it? Why and how might you use it?

**DOI:** 10.1186/s12909-017-1115-9

**Published:** 2018-01-05

**Authors:** Jon Dowell, Jennifer Cleland, Siobhan Fitzpatrick, Chris McManus, Sandra Nicholson, Thomas Oppé, Katie Petty-Saphon, Olga Sierocinska King, Daniel Smith, Steve Thornton, Kirsty White

**Affiliations:** 1 School of Medicine Deanery, Ninewells Hospital & Medical School, Dundee, DD1 9SY UK; 2 Institute of Education for Medical and Dental Sciences, University of Aberdeen, West Wing, Polwarth Building, Foresterhill, Aberdeen, AB25 2ZD UK; 3 Medical Schools Council, Woburn House, 20 Tavistock Square, London, WC1H 9HD UK; 4 Psychology and Medical Education, University College London, Gower Street, London, WC1E 6BT UK; 5 Student Progression & the Centre for Medical Education, Barts and the London School of Medicine and Dentistry, QMUL, Room 2.43 Garrod Building, Turner Street, London, E1 2AD UK; 6 General Medical Council, Regents Place, 350 Euston Road, London, NW1 3JN UK; 7 Health & Obstetrics, Barts and the London School of Medicine and Dentistry, QMUL, VP (Health) Offices, 2nd Floor, Dean Rees House, Charterhouse Square, London, EC1M 6BQ UK

**Keywords:** Selection, Personnel, Medical schools, Education, Medical, Graduate, Education, Medical, Undergraduate, Fitness to Practise

## Abstract

**Background:**

Educating doctors is expensive and poor performance by future graduates can literally cost lives. Whilst the practice of medicine is highly evidence based, medical education is much less so. Research on medical school selection, undergraduate progression, Fitness to Practise (FtP) and postgraduate careers has been hampered across the globe by the challenges of uniting the data required. This paper describes the creation, structure and access arrangements for the first UK-wide attempt to do so.

**Overview:**

A collaborative approach has created a research database commencing with all entrants to UK medical schools in 2007 and 2008 (UKMED Phase 1). Here the content is outlined, governance arrangements considered, system access explained, and the potential implications of this new resource discussed. The data currently include achievements prior to medical school entry, admissions tests, graduation point information and also all subsequent data collected by the General Medical Council, including FtP, career progression, annual National Training Survey (NTS) responses, career choice and postgraduate exam performance data. UKMED has grown since the pilot phase with additional datasets; all subsequent years of students/trainees and stronger governance processes. The inclusion of future cohorts and additional information such as admissions scores or bespoke surveys or assessments is now being piloted. Thus, for instance, new scrutiny can be applied to selection techniques and the effectiveness of educational interventions. Data are available free of charge for approved studies from suitable research groups worldwide.

**Conclusion:**

It is anticipated that UKMED will continue on a rolling basis. This has the potential to radically change the volume and types of research that can be envisaged and, therefore, to improve standards, facilitate workforce planning and support the regulation of medical education and training. This paper aspires to encourage proposals to utilise this exciting resource.

## Background

Medicine is a cornerstone of higher education globally, with high financial cost and academic resource requirements. Whilst there is no shortage of applicants, there is debate over equity of access, diversity and workforce requirements as well as other issues. In response, the UK Government announced in 2017 an additional 1500 (over 20%) medical school places [1] with the objective of producing graduates interested in less popular specialities and prepared to work with remote or deprived communities.

In the UK, it is estimated the current 7800 medical school entrants cost the state around £180,000 each (plus personal living expenses) to complete their primary medical qualification. However, this investment of approximately £1.5bn per annum has no organised research and development arm [2]. Furthermore, the data on which to base selection decisions have never been systematically gathered, which explains why there has been a consistent paucity of robust longitudinal studies within medical education [3–7]. Lack of such UK wide studies was identified as a weakness in the field’s scholarly output by the 2014 UK Research Excellence Framework [8–10]. Rare events such as leaving medicine or Fitness to Practise (FtP) concerns, require collaboration to collate sufficient numbers for evaluation, and are particularly challenging to perform. The evident success of the multiple UK Birth Cohort Studies is encouraging but also testifies to the challenges of maintaining such as resource [11–13].

This paper outlines the concept as well as current and planned content of a novel UK national medical education research database (UKMED), and invites researchers and educationists internationally to consider how they might use it. The potential value of a mechanism for tracking the progress of students through medical school and into postgraduate practice, enabling a wide range of original studies to be conducted, has been recognised and is not without precedent. Collating and integrating such a large-scale database could enable high quality longitudinal studies to address significant research questions ranging from selection, through under- and postgraduate training, and eventually into clinical practice and patient outcomes.

Within the UK, the General Medical Council (GMC) has a statutory function under the Medical Act 1983 to co-ordinate the stages and promote high standards of medical education [14]. A database linking educational outcomes gives the potential to explore the effect of policy changes at each stage of training (medical school, foundation school and postgraduate training programmes) independently. This is key as the GMC’s statutory function justifies using personal data about students and doctors in compliance with the Data Protection Act.

We are not the first to work towards these goals and internationally there have been four related initiatives that we are aware of:

- The UK Medical Careers Research Group (MCRG) undertook sequential studies of graduate cohorts from 1973 leading to over 100 publications [15]
- McManus [16] has led a series of cohort studies, mostly from St Mary’s Hospital Medical School (now part of Imperial College), spanning many years and leading to multiple high impact outputs [17, 18]
- The Medical Schools Outcomes Database was devised for workforce planning and sought to track graduates in Australia and New Zealand, for example predicting who might wish to work rurally [19, 20]
- Jefferson Medical School has tracked its own graduates since 1964, leading to a highly effective research programme [21].

There are also multiple pre-admissions testing organisations that have an interest in evaluating the validity and utility of their assessments and have collaborated with academics in a range of studies. They have provided important insights, especially in USA and Canada, though typically, these organisations have been restricted to a limited range of relatively short-term outcomes (e.g. licensure exam data) or a small number of medical schools [22]. However, even for non-profit making admissions test providers, funding research could be considered to potentially introduce bias.

Key examples of work to evaluate the validity and utility of pre-admissions testing are available from:

- The UK Clinical Aptitude Test (UKCAT) [23]
- Graduate Medical School Admissions Test (GAMSAT) [24]
- The Health Professions Admission Test (HPAT) [25]
- Undergraduate Medicine and Health Sciences Admission Test (UMAT) [26]
- The Medical College Admission Test® (MCAT®) [27]

The UKCAT consortium commenced in 2005, and since 2006 around 20,000 applicants have sat this aptitude test each year. From its inception, the research potential of data on this scale was apparent but proved hard to realise, primarily because of data protection concerns. It took until 2012 for the first UK wide analyses to emerge [28] since when over 19 UKCAT related studies have been published [23]. The consortium has enabled innovative approaches such as the UKCAT-12 study [29, 30], and a relevant proof of concept UG-PG matching study [31].

Hence, in 2011, the Medical Schools Council (MSC) and the GMC were asked to consider extending this database into a comprehensive and ongoing tracking system and research resource, which would link pre-admission metrics (e.g. performance in school level qualifications such as A-levels or Scottish Highers and performance on admissions tests like UKCAT), through graduation and into postgraduate careers. This wider database now also enables new and more detailed areas of research such as:

- Recruitment and selection in terms of equity of access and impact on graduate qualities and workforce issues
- Assessing the impact of variation or changes in undergraduate education such as comparing graduate and direct entry systems or traditional/integrated/problem-based curricula
- Assessing variation in qualities of graduates using a range of outcome measures such as specialist postgraduate exam performance, career choice or FtP events
- Equality and diversity in terms of access to and performance within medical careers
- Workforce planning and career progression
- Patient safety and FtP events
- Improving all studies by enabling multivariate analysis to adjust for confounding variables, in particular prior academic attainment.

While no single approach can address all the issues, in this commentary we introduce UKMED as an innovation that offers the opportunity to better understand many of these complex dilemmas. Because patterns of background, performance and capability as a medical student, trainee or doctor often have international relevance and present issues common to all countries, researchers anywhere are invited to utilise UKMED’s unique potential.

## Schema and implementation

### How was UKMED piloted?

**Fig. 1:**
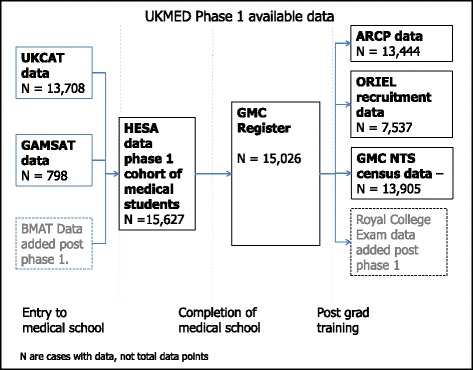
UKMED Phase 1 available data

The UK Medical Education Database Phase 1 (2015–2016) was a collaboration that achieved the acquisition, linkage, governance and access to a broad range of routine data on all entrants to every UK medical school (*N* = 15,627) in 2007 and 2008. Extensive data were gathered from the point of application onwards, including graduation, the GMC’s National Training Survey (NTS) and career progression (see Fig. 1). Working across agencies enabled three ‘proof of concept’ longitudinal studies to be conducted. The intention was to establish a resource that could be expanded with the addition of successive cohorts and further datasets, ultimately including the planned UK national Medical Licensing Assessment which should provide a common academic outcome measure [32].

UKMED Phase 1 established complete coverage of UK medical school entrants and started to support multiple studies. See the UKMED website [33] for details of all approved studies, data dictionary including online coverage tool, and application forms.

The process has required extensive consultation and legal guidance to address data protection, management and academic governance issues. It was supported by joint leadership from the GMC and MSC, which, due to their roles with all UK medical schools, created a willingness to review and address issues such as data sharing agreements; privacy notices and establishing the GMC as the ‘Data Controller’. (As the Data Controller, the GMC has responsibility for ensuring compliance with the Data Protection Act.) This process took two years but overcame many hurdles, including concerns regarding Freedom of Information (exempt as a research database) and universal coverage as, by using HESA data, UKMED is able to include all UK students in established and emerging medical schools and including UKCAT and GAMSAT selection tests where relevant. Important limitations are acknowledged and discussed below.

### Initial database content

In terms of structure based on Phase 1, HESA entry data defined cases for inclusion in UKMED (as not all those who start medical degrees progress to registration) and were linked to test provider data from UKCAT, and GAMSAT using the UCAS person ID [34, 35]. Graduates were matched to the GMC register using the medical school code and medical schools’ internal identifying number for each of their students [36], which the GMC receives as part of the provisional registration process, providing an efficient and reliable approach. There were no selection biases – all cases were included. The GMC number provides links to postgraduate data, including Annual Review of Competence Progression (ARCP) outcomes and royal college exam results; a full list is in the UKMED data dictionary [37].

### Developments since the phase 1 pilot

**Fig. 2:**
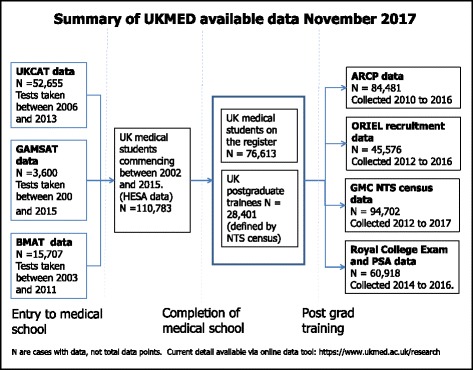
Summary of UKMED available data November 2017

UKMED is a live project and the UKMED website outlines the current data available, approved research projects and their status [33]. Key developments since the successful completion of Phase 1 are outlined in Fig. 2 and described below.

The UKMED population is now defined in two ways:

- All those who started at a UK medical school since 2002 as defined by the HESA data (*N* = 110,78). The GMC has obtained historical data and now receives updates annually.
- Those who have taken part in postgraduate training in the UK since 2012 as captured by the GMC annual census for the National Training Survey [38]. This includes trainee doctors who obtained their primary medical qualification outside of the UK (*N* = 42,490). Inclusion of all doctors in postgraduate training allows UKMED to be used for studies looking at the predictive validity of selection methods used for postgraduate training programmes. It may also increase the opportunity for international comparisons.

### Additional data

Since piloting, UKMED has moved to an annual cycle collating a broader range of undergraduate and test-provider data as well as postgraduate performance and exam data. UKMED now includes:

- Data from all medical royal and faculty exams sat from 1 August 2013 by any GMC registered doctor, updated annually
- Data since 2014 from the UK Prescribing Safety Assessment (PSA) [39]
- BMAT scores from 2003 [40]
- Data from the Multi-Specialty Recruitment Assessments used for postgraduate training programme selection
- Data on practice history. GMC data collected for revalidation purposes originally provided from payroll systems by the four departments of Health: ESR – Electronic Staffing Records. PCIS – Primary Care Information System and SWISS – Scottish Workforce Information Standard System. This allows cases to be tracked through to post-training employment.

### Enhancements to the governance processes

It is now possible for researchers to include data generated themselves in a UKMED research extract. This is subject to an information governance review to confirm legality and the presence of suitable identifier for linking purposes. Researchers doing this must make the data available to others via UKMED following completion of their study.

## Utility and discussion

### Access

UKMED provides access to matched data via a safe haven for studies approved on the basis of their academic rigour and value [41]. This approach helps address a number of privacy concerns that have hampered research using linked data in other contexts [42]. It can only be accessed by application to ensure due diligence. Applications are reviewed by an expert panel against the publicly available criteria, including confirmation that only appropriate data are requested. On the basis of this review, a recommendation on each application is made by the UKMED Advisory Board to the GMC as data controller. There are two meetings a year at which applications are reviewed.

The GMC ensures compliance with the Data Protection Act by de-identifying the data: cases are assigned their own unique Study–Id and quasi-identifiers are recoded so unique cases cannot be identified in the extract [43]. The safe haven further minimises the risk of re-identification; allowing the researchers to run analyses on the extract using the statistical packages of their choice, whilst preventing the export/import of data and re-identification through linkage. Researchers are under contract to use the data only for the purposes of the approved proposal. Analytic outputs are reviewed to ensure compliance with HESA statistical disclosure controls [44] prior to release to researchers, and all reports are screened prior to publication.

Current guidance from the NHS Health Research Authority [45] states that Research Ethics Committee (REC) permission is not required, as two exemptions are applicable to UKMED [46].

“Research limited to secondary use of information previously collected in the course of normal care (without an intention to use it for research at the time of collection) is generally excluded from REC review, provided that the patients or service users are not identifiable to the research team in carrying out the research.”

“Research involving staff: REC review is not normally required for research involving NHS or social care staff recruited as research participants by virtue of their professional role.”

This exemption only applies to data held exclusively in UKMED, so studies that introduce external data may need separate ethical approval and researchers may be required to obtain this from their local committee.

The GMC’s Information Governance Team reviews the privacy statements shown to data subjects; if data collection for a study has not yet commenced, UKMED recommends privacy notices make it clear that:

- Identifiable data may be used for future research
- Identifiable data may be shared with third parties to undertake the research.

### Interface

Researchers access data by logging onto the safe haven portal provided by the University of Dundee Health Informatics Centre (HIC) [47]. Once logged in remotely to the safe haven they are able to work using a Windows desktop and a range of statistical packages. Results are saved onto an output directory which is reviewed before sending to the researcher. Full details are described in the *Process for completing UKMED research* document [41].

### Cost

It is anticipated that access will continue to be provided free of charge. When researchers wish to link additional data to UKMED, they may be asked to cover associated costs, with requests reviewed on a case-by-case basis.

### Developments

The creation, use and interpretation of prospective databases is complex. As Pearson [11] eloquently describes, it is impossible to predict the creative unexpected uses that tend to emerge over time, nor fully address the challenges presented. In particular, the difficulties managing missing data, defining socioeconomic class and equating prior academic attainment of students are challenges within UKMED. A project is underway amongst those conducting the early studies to create some UKMED standard approaches that can be used to simplify these issues. For instance, a syntax for calculating select derived variables using consistent methods is now available and a common approach for multiple imputation of missing values is being considered.

### Utility

Details of accepted research proposals and their status are available on the UKMED website [33]. In future, UKMED could enable:

- The impact of selection tools to be evaluated in far greater detail and against a wide range of important outcome markers. The comprehensive scale and coverage allows for complex subgroup analysis exploring the impact of background and prior attainment as well as comparing selection tests. Access to retention data, common assessments such as the Situational Judgment Test for selection to the UK Foundation Programme and the PSA introduces new opportunities. Career choices, progression and postgraduate exam performance can be assessed and evaluated in the light of prior attainment and background
- Validation studies in the event of the introduction of a UK Medical Licensing Assessment [32]
- Studies into FtP information at the point of graduation, ARCP data and speciality selection information. Thus, an entirely new set of information has been made available for vital key (and relatively rare) performance outcome markers
- Studies employing new fields in existing datasets, for instance the GMC’s annual NTS can be amended to include additional questions.
- Studies linking external data, for example some centres already have data that merit inclusion (Multiple Mini Interview scores or Conscientiousness Index) and UKMED can be used to both improve and expedite the assessment of these emerging tools. Indeed, entirely new tools can now be designed, and data banked in anticipation that UKMED will provide a follow up mechanism in due course. In particular, this might apply to novel non-academic selection or assessment measures.

Finally, it is possible to conceive of ways in which UKMED might inspire not only new comparisons but generate new interventions. Medical schools could collaborate on testing alternative approaches to complex issues such as improving graduates’ resilience. Even cluster randomised trials might be considered feasible now an efficient follow up system is in place.

### The limitations of UKMED.

The data in UKMED are administrative, collected by routine systems. Such data describe *what* happened but not *why* or *how*. There are situations within medical education where qualitative data are very informative. Questions such as unequal access to medical school, the reasons why graduates are reluctant to enter some specialities or work with remote or deprived communities, and doubts about the wider impact of different approaches to selection or education would benefit from qualitative or mixed methods approaches. At the medical school level, there is little data on student aspirations, motivations, interests, personalities and a host of other individual differences which probably underpin much variation. However, UKMED may in future collect some qualitative data in terms of ‘white space boxes’, for instance via the NTS or other independent surveys.

A further limitation concerns examination results. UKMED is collecting data on overall performance in selection tests, medical school examinations (in the form of selection scores for the Foundation Programme) and postgraduate examinations. However, those are total scores of individual applicants. Answers to individual items might be of interest but would produce an extremely complex dataset. Limited data on performance during the undergraduate course are available from some medical schools.

A different sort of limitation is that UKMED only collates data on individuals admitted to medical schools. At present, it does not collect data on individuals who applied but did not enter any UK school, limiting potential for research into selection processes and introducing range restriction issues. For example, if investigating the relationship between UKCAT scores and demographic variables only the better performing cases would be available for analysis within UKMED (such studies would be better conducted using the UKCAT database). Furthermore, even those who apply for medical school are a limited subset of the population as a whole. Recent work suggests that about 10% of 12-year olds put medicine as their first choice for a future career, a figure far removed from the 1% or so of individuals who eventually become doctors [48]. Finding out about early self-selection is not easy, but might be possible if UKMED can be linked in future to large cohorts such as the Millennium Cohort Study [49].

## Conclusions

The UKMED educational research database presents unique opportunities for multicentre longitudinal studies on ‘big numbers’ covering complex questions. Several studies have been completed and submitted for publication. Although based on UK students, the results have direct relevance for many countries. Research applications for access to datasets are not limited to those in the UK. The challenge is now to ensure that the medical education community takes full advantage of this outstanding new resource.

## Abbreviations

AoMRC: Academy of Medical Royal Colleges
ARCP: Annual Review of Competence Progression
BMAT: Biomedical Admissions Test
CK: Clinical Knowledge
FPAS: Foundation Programme Application System
FtP: Fitness to Practise
GAMSAT: Graduate Medical School Admissions Test
GMC: General Medical Council
HEE: Health Education England
HESA: Higher Education Statistics Agency
HIC: Health Informatics Centre, University of Dundee
HPAT: The Health Professions Admission Test
MCAT: Medical College Admission Test
MCRG: UK Medical Careers Research Group
MSC: Medical schools council
NTS: National Training Survey
ORIEL: UK wide portal for recruitment to postgraduate medical training programmes.
PSA: Prescribing Safety Assessment.
REC: Research Ethics Committee.
SJT: Situational Judgement Test.
UKFPO: United Kingdom Foundation Programme Office.
UKCAT: UK Clinical Aptitude Test.
UKMED: United Kingdom Medical Education Database.
UMAT: Undergraduate Medicine and Health Sciences Admission Test.
